# Comprehensive Structure of the Female Marine Water-Strider *Asclepios annandalei* Distant, 1915 from Pranburi River Estuary, Thailand: New Information for the Genus *Asclepios*

**DOI:** 10.21315/tlsr2022.33.3.4

**Published:** 2022-09-30

**Authors:** Pisit Poolprasert, Sinlapachai Senarat, Jes Kettratad, Gen Kaneko, Ezra Mongkolchaichana, Natthawut Charoenphon, Narit Thaochan

**Affiliations:** 1 Faculty of Science and Technology, Pibulsongkram Rajabhat University, Phitsanulok, 65000 Thailand; 2 Department of Marine Science and Environment, Faculty of Science and Fisheries Technology, Rajamangala University of Technology Srivijaya, Trang Campus, Sikao, Trang 92150, Thailand; 3 Department of Marine Science, Faculty of Science, Chulalongkorn University, Bangkok 10330 Thailand; 4 College of Natural and Applied Science, University of Houston-Victoria, Victoria, Texas 77901, USA; 5 Department of General Education, Faculty of Science and Health Technology, Navamindradhiraj University, Bangkok 10300 Thailand; 6 Department of Anatomy, Faculty of Medical Science, Naresuan University, Phitsanulok 65000 Thailand; 7 Agricultural Innovation and Management Division (Pest Management), Faculty of Natural Resources, Prince of Songkla University, Hat Yai, 90110 Thailand

**Keywords:** Gerridae, Microanatomy, Organs, Thailand, Water-Strider Insect

## Abstract

The objective of this study was to describe the structure and histochemistry of the systemic organs in the female marine water-strider *Asclepios annandalei* from Pranburi river estuary, Thailand. Results from this study revealed for the first time that the integumentary system of this species consisted of three layers including epicuticle, exocuticle and endocuticle. The muscular system apparently contained only skeletal muscle along the body. In the urinary system, we observed well-developed Malpighian tubules, each of which was covered with the simple cuboidal epitheliums. These epitheliums also contained the secretory granules that were reacted positively with periodic acid Schiff (PAS). The digestive system of this species was composed of three distinct parts including foregut, midgut and hindgut. The respiratory system was composed of the respiratory organ, which was rarely found near the integument system. This organ was lined with a simple squamous epithelium. Two regions of nervous system, i.e., frontal ganglion connected to the eye structure and ventral nerve cord, were found. Each ganglion basically consisted of two layers, outer cortex and inner medullae. The outer cortex contained three types of cells, including neurosecretory cells, neuroglial cells and neurons. The cytoplasmic inclusion of neurosecretory cells contained secretory granules, which reacted positively with PAS, indicating the presence the glycoprotein. The neuroglia and neuron were also observed in the inner medullae layer. The female reproductive system (the ovarian structure, the reproductive tract and the accessory organ) of this gerrid species was seen under histological sections. The well-developed integument system and Malpighian tubule as well as the abundant respiratory organ is a characteristic of this species, which might be useful for the adaption to the estuarine condition.

**Highlights**
- The systemic organs in the female marine water-strider, *Asclepiosannandalei* from Pranburi river estuary, Thailand were originallyreported in terms of structure and histochemistry.
- Along the body, the muscular system of marine water-strider, *Asclepios annandalei* appeared to be limited to skeletal muscle.
- This marine water-strider (*A. annandalei*) has traits including awell-developed integument system, Malpighian tubule, and anabundance of respiratory organs that may help it adapt to the estuarine environment.

## INTRODUCTION

The Genus *Asclepios* is commonly considered as a sea skater which is found primarily in the brackish water of mangrove rather than coral reefs or open seas. There are three well-known species in this genus: *Asclepios shiranui* Esaki, 1924; *A.apicalis* Esaki, 1924 and *A. annandalei* Distant, 1915, all of which have been reported only from East and Southeast Asia (Andersen & Cheng 2004; Sites& Vitheepradit 2010). *A. shiranui* is listed as an endangered species due to its very restricted distribution along the Western or Southwestern coasts of Japan(Ikawa *et al*. 2012; Environment Agency 1991), whereas *A. apicalis* is commonly observed in Taiwan and Vietnam (Andersen & Cheng 2004). *A. annandalei* has been reported only in Malaysia, Singapore, Sri Lanka and Thailand (Andersen &Cheng 2004; Sites & Vitheepradit 2010).

Several previous studies have provided basic biological characteristics of *A. annandalei* such as taxonomy, ecology, morphology and phylogeny (Andersen& Foster 1992; Cheng *et al*. 2001; Damgaard *et al*. 2000; Polhemus & Cheng 1982). However, comprehensive structural information of this species has not been reported. Detailed morphological and histological data will facilitate new approaches from the viewpoint of physiology, histopathology and biochemistry, which will increase our overall knowledge about the Genus *Asclepios* and family Gerrinidae. It will also help us to answer the key question that potentially contributes to the conservation of this species – How can *A. annandalei* live under a harsh estuarine environment where salinity changes continuously? Here, we determined the systematic structure and histochemistry of *A. annandalei* female in Pranburi river estuary, Thailand, via histological and histochemical techniques.

## MATERIALS AND METHODS

### Water-Strider Insect Sampling

A total of fixed 20 mature female *A. annandalei* were obtained from the Fish Biology and Aquatic Health Assessment (FBA-LAB), Department of Marine Science, Faculty of Science, Chulalongkorn University, Thailand. These insects were field collected as a voucher specimen during the sampling of estuarine fish from Pranburi river estuary, Thailand (N 12°24′8.5″/E 99°59′0.2″). The total lengths of these specimen ranged from 1.0 cm–1.2 cm.

### Histological Techniques

All samples were processed as whole mount according to standard histological protocols (Presnell & Schreibman 1997; Suvarna *et al*. 2013). The paraffin block was cut at a thickness of 4 μm using a rotary microtome. The sections were stained with a solution of Harris's hematoxylin and eosin (H&E) to assess histological structure and components, Masson's Trichrome (MT) to observe connective tissue/fibres and Periodic acid-Schiff-hematoxylin (PAS-H) to assess glycoproteins. Histological slides were viewed and photographed with a Leica TE750-U (Boston Industries, Inc., USA).

## RESULTS AND DISCUSSION

**Figure 1:**
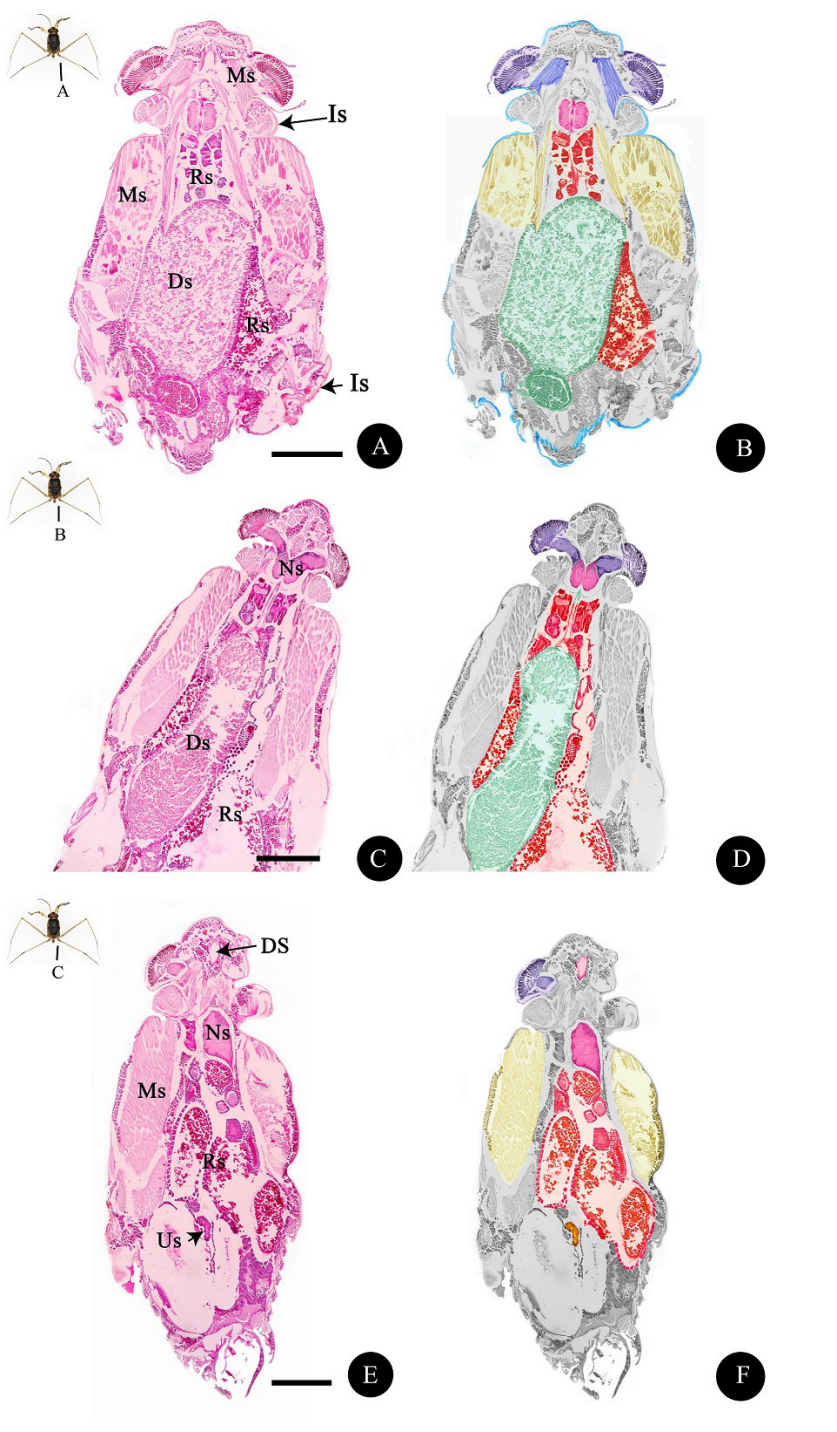
Light photomicrographs (A, C, E) and schematic diagrams (B, D, F) of the *A. annandalei* integument system (Is, blue in schematic diagrams), muscular system (Ms, yellow), digestive system (Ds, green), reproductive system (Rs, orange), urinary system(Us) and nervous system (Ns) composed of two regions: dorsal horn and eye (purple) and ventral horn (pink) (A–F). *Notes*: Scale bar A, C, E = 500 μm. Harris's hematoxylin and eosin (H&E), Masson's Trichrome (MT) and Periodic acid-Schiff-hematoxylin (PAS-H).

Observation of whole-mount *A. annandalei* under the light microscopy allowed us to classify structural organisations of *A. annandalei* into multiple distinct systems based on localisation, organ properties, tissue/cell compositions and staining patterns. The overall schematic diagrams are shown as Fig. 1. Details of each system are described in each section.

### Integumentary and Muscular Systems

**Figure 2:**
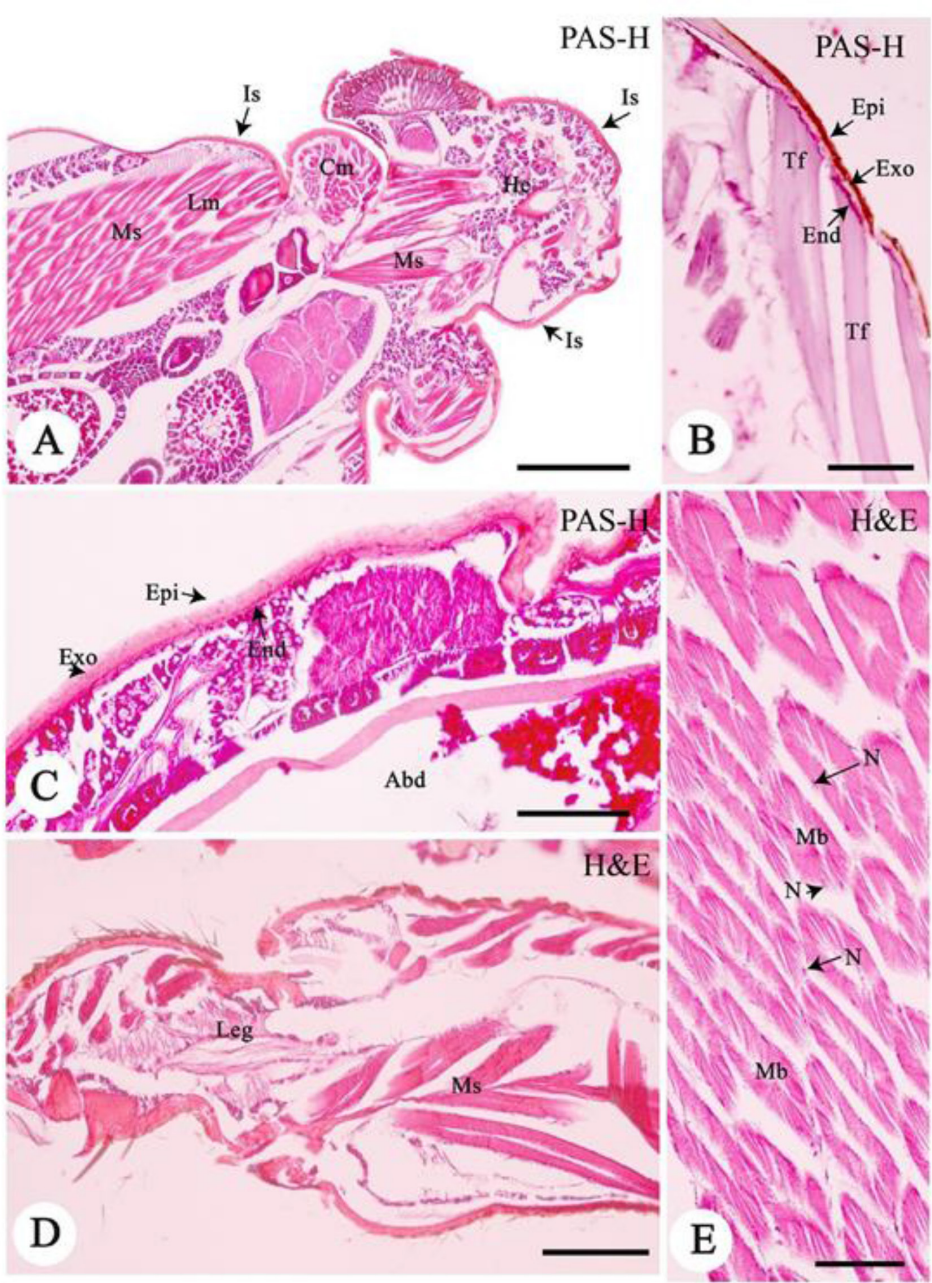
Light photomicrographs of the integument system (Is) and muscular system (Ms) of *A. annandalei*. (A) The muscular system consisted of two types of muscles [longitudinal muscle (Lm) and circular muscle (Cm)]; (B–C) Integument system (Is) contained three distinct types of layers: epicuticle (Epi), exocuticle (Exo) and endocuticle (End); (D–E) Muscle bundles. *Notes*: Abd = abdomen, He = head, Leg = leg, Mb = muscular bundle, N = nucleus, Tf = tonofilament. Scale bar A = 200 μm, D = 50 μm, B, C, E = 20 μm. Harris’s hematoxylin and eosin (H&E), and Periodic acid-Schiff-hematoxylin (PAS-H).

The integumentary system was observed throughout the body (Figs. 2A and 2B). The integumentary system had three basic layers, epicuticle, the exocuticle, and the endocuticle from outside to inside (Fig. 2B, PAS method). The epicuticle was a thick surface layer of brown colour. Occasionally, this structure was missing in the head and abdomen integuments, perhaps resulting from an artifact during the histological processing (Fig. 2C). The exocuticle was the middle layer thicker than other integument layers (Figs. 2B and 2C). The exocuticle layer was eosinophilic and had pink colour in both H&E and PAS methods, indicating the presence of glycoproteins (Figs. 2B and 2C). The endocuticle was seen as a thin line of dark pink under the light microscopy (H&E and PAS methods) (Figs. 2B and 2C).

The skeletal muscles were observed in circular and longitudinal muscle regions along the lateral side of the body, especially in the abdominal region and legs (Figs. 2A and 2D). This muscle consisted of eosinophilic muscle bundles (Fig. 2E, H&E method). Longitudinal sections revealed that the muscle bundles have several muscle fibers (or muscle cells) due to their positive reactions in the MT method (greenish colour, data not shown). An oval nucleus was heterochromatic and displaced, i.e., located peripherally in muscle cells (Fig. 2E). These results did not differ from those reported from *Epicauta waterhousei* (Langkawong *et al*. 2013) 49 and *Oligotoma saundersii* (Poolprasert & Senarat 2014). Between the skeletal muscles and the cuticle, the tonofilament was also found along the abdomen (Fig. 2B).

### Excretory System

**Figure 3:**
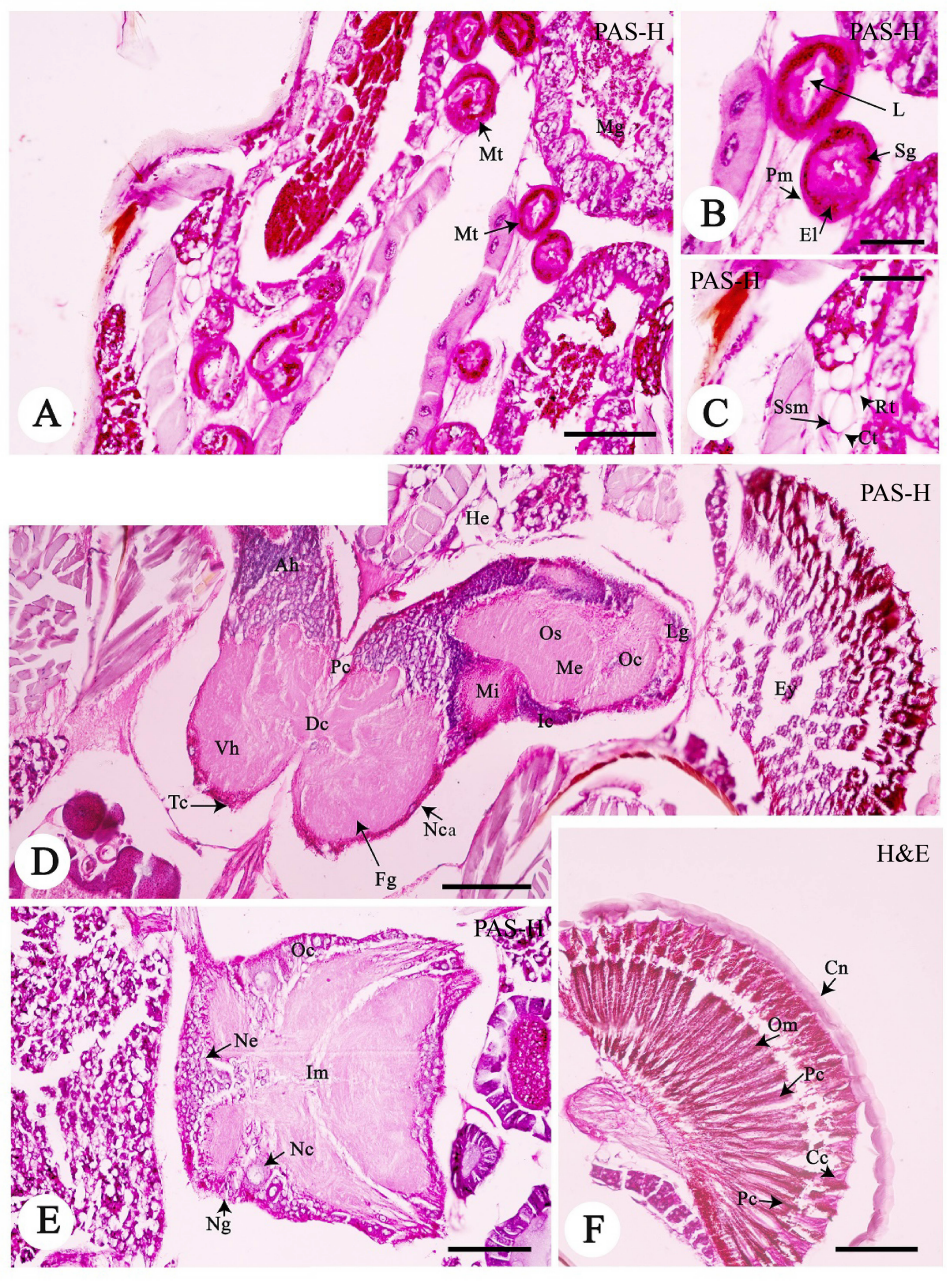
Light photomicrographs of (A–B) the urinary system, (C) respiratory system and (D–F) nervous system of *A. annandalei*. *Notes*: Ah = anterior horn, Cc = crystalline cone, Cn = cornea, Ct = connective tissue, DC = deutocerebrum, El = epithelial layer, Ey = eye, Fg = frontal ganglion, He = head, Ic = inner of optic chiasma, Im = inner medulla, L = lumen, Lg = leg, Me = medulla externa, Mg = midgut, Mi = medulla interna, Mt = mulpighian tubules, Nc = neurosecretory cell, Nca = neuronal capsule, Ne = neuron, Ng = neuroglia, Oc = outer of optic chiasma, Om = ommatidium, Os = optic stalk, Pc = protocerebrum, Pm = peritoneal membrane, Rt = respiratory tubule, Sg = secretory granule, Ssm = simple squamous epithelium, Tc = tritocerebrum, Vh = ventral horn. Scale bar A = 50 μm, D = 50 μm, E, F = 20 μm, B, C = 10 μm. Harris’s hematoxylin and eosin (H&E), and Periodic acid-Schiff-hematoxylin (PAS-H).

Malpighian tubule is one of the major parts of the excretory system (Fig. 3A). Note that it seemed to predominate in several locations between midgut and hindgut (Fig. 3A). The tubular lumen was embedded in a homogenous eosinophilic matrix (PAS method) (Fig. 3B). The tubule contained two layers, the epithelial layer and peritoneal membrane (Fig. 3B). The epithelial layer was lined with simple cuboidal epithelium (Fig. 3B). These cells had the centrally located euchromatic nuclei, and the cytoplasm were strongly basophilic in the H&E method. The epithelial features were similar to those of other insects; for example, Coleoptera (Areekul 1957), Lepidoptera (Standlea & Yonke 1968) and Hymenoptera (Arab & Caetano 2002). Interestingly, the secretory granules were also observed in the cytoplasm, as demonstrated by positive staining in the PAS method (Fig. 3B), although the role of these secretory granules are unknown. A possible function may be protein synthesis to support the function of excretory system by, for example, absorbing water and nitrogenous wastes (uric acid) from the hemolymph and excreting the undigested food materials via the anus (Berridge & Oschman 1969). Several previous observations showed that Malpighian tubules are the main osmoregulatory organ and are considered analogous to the nephridia of annelids and the kidneys of vertebrates (Snodgrass 1956; Wigglesworth 2003). The activity of a co-transport system and a H^+^ pump in the Malpighian tubules of *Formica polyctena* has been experimentally recorded (Leyssens, Zhang, *et al*. 1993; Leyssens, Van Kerkhove, *et al*., 1993). It is interesting to hypothesise that the well-developed Malpighian tubule of *A. annandalei* may be related to the adaptation to estuarine environment. The peritoneal membrane was externally lined by a thin layer of connective tissue (Fig. 3B).

### Respiratory System

Although two distinct histological features, the spiracles and the trachea, have been recorded as the respiratory system of insects in the literature (Borror *et al*. 1989; Gullan & Cranston 2004), these features were not identified in *A. annandalei* under light microscopy. Hence, we call the respiratory organs of this species as “the respiratory tubules”, which were scattered between the thorax and the abdomen (Fig. 3C). The respiratory tubules have been reported from other species such as *Popilius disjunctus* (Robertson 1962) and *Tetraponera rufonigra* (Somala *et al*. 2020). The respiratory tubules of *A. annandalei* female were small and had oval-spherical shapes. These tubules were rarely observed (or a lesser amount) in the body cavity. Each tubule was covered by simple squamous epithelium and surrounded by a very thin layer of connective tissue (Fig. 3C). This system plays a role in regulating (uptake) oxygen from the air and eliminated carbon dioxide resulting from cellular respiration in the tissues to the air (Borror *et al*. 1989; Gullan & Cranston 2004; Hetz & Bradley 2005; Weibel 1984).

### Nervous System

The central nervous system of *A. annandalei* consisted of two distinct parts; the brain and ventral nerve cord similar to previous observations of insects (Adams & Selander 1979; Gullan & Cranston 2004). The frontal ganglion was situated at the central part of the head. As shown in the longitudinal section (Fig. 3D), the frontal ganglion was embedded within the cranial capsule, being covered by a thin of connective tissue (or neuronal capsule). It was composed of anterior and ventral horns (Fig. 3D). The anterior horn constituted two components including the protocerebrum and the deutocerebrum, whereas the ventral horn contained the ventral tritocerebrum (Fig. 3D). In particular, to the lateral protocerebrum (or corpora pedunculata or mushroom bodies), there was paired lobes to join the eye structure. This region was divided by the optic nerve (or optic stalk). At high magnification, the optic nerve was classified into three regions (a medulla interna, amedulla externa, and an inmina gabglionaris) (Fig. 3D).

In the ventral nerve cord, two ganglia [sub-ealoesophageal ganglion (Fig. 3E) and abdominal ganglion (Data not shown)] were observed. Each ganglion was similarly structured and composed of two layers: the inner medullae and the outer cortex (Fig. 3E). The inner medullae constituted the nerve fibres and the neuroglia (Fig. 3E). The outer cortex had abundant multilayers of neuronal cells, in which they were classified into three types according to sizes and histologically characterisations.

1. The oval-shaped neurosecretory cell (Nc), which was the largest (about 5–6μm in diameters) among the three cell types. Nc had an oval nucleus containing one or two central nucleoli. Nc was also surrounded by an eosinophilic nucleoplasm (Fig. 3E).
2. The oval-shaped neuronal cell of about 3–4 μm diameters.
3. The neuroglia with the smallest size of about 2 μm diameter. This cell was generally located among other types of cells (Fig. 3E). It had a round nucleus and surrounded by a rim of the eosinophilic cytoplasm.

Sagittal sections of the paired compound eyes of *A. annandalei* were observed (Fig. 3F). The ommatidia showed a regular pattern similar to closely packed facets (Fig. 3F). Each ommatidium had an elongated shape, which was divided into two zones: the outer and the inner zones. The outer zone consisted of a transparent bi-convex cornea of the eye (Fig. 3F). The inner zone appeared under the cornea structure. Several cell types including the crystalline cone and the photoreceptor cells (or retinular cells) were observed throughout the ommatidium. Each ommatidium was separated by rhabdomere. In addition, the pigment cell of an elongated contained several brown pigments and was located among the ommatidium (Fig. 3F).

### Digestive System

**Figure 4:**
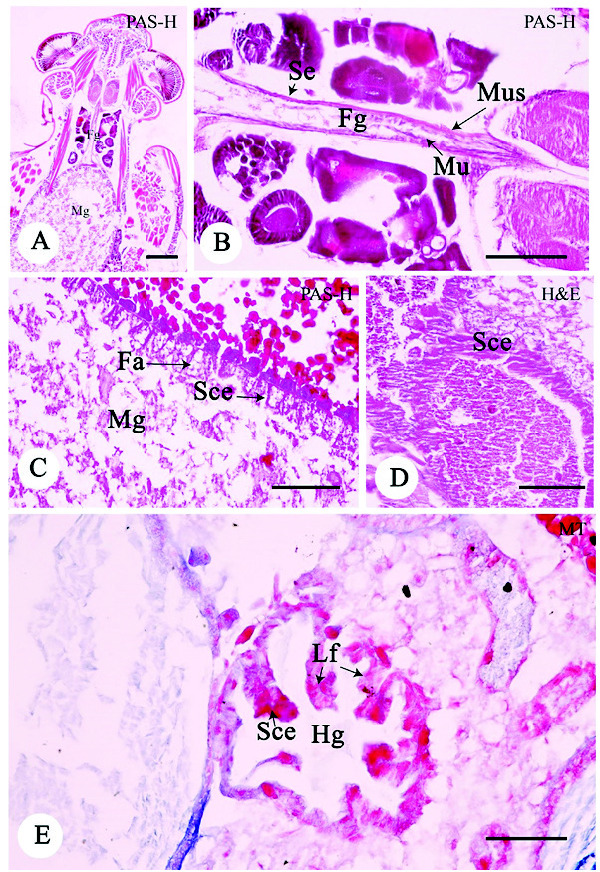
Light photomicrographs of the digestive system of *A. annandalei* composed of three regions including Fg (A–B), Mg (C–D) and Hg (E). *Notes*: Fa = foamy appearance, Lf = longitudinal fold, Mu = mucosa, Mus = muscularis, Sce = simple cuboidal epithelium, Se = serosa. Scale bar A = 100 μm, B = 50 μm, E = 20 μm, C, D = 10 μm. Harris’s hematoxylin and eosin (H&E), Masson's Trichrome (MT) and Periodic acid-Schiff-hematoxylin (PAS-H).

In the long-sectional view, the digestive system of *A. annandalei* was divided into three parts based on the localisation and histological structure: foregut, midgut, and hindgut (Fig. 4). The foregut was a slender tubule composed of three layers from the inside to the outside: mucosa, muscularis and serosa (Figs. 4A and 4B). The mucosal layer was lined with a very thin simple squamous epithelium without microvilli structure (Fig. 4B), which is similar to the finding in *Sesamia calamistis* (Grice & Durr 1969). The major muscularis contained connective tissues around the foregut (Fig. 4B). The midgut was a circular-shape organ and the largest part in the digestive system (Fig. 4C), in which the mucosal layer was lined with the non-ciliated low simple cuboidal epithelium (Fig. 4C). The prominent characters of each epithelium include empty vesicles of foamy appearance (Fig. 4C). The structure of other layers was similar to that of the foregut. The mucosal hindgut was formed into the longitudinal fold (Fig. 4E), which was lined with the simple cuboidal epithelium (Fig. 4E). The data that the mucosal midgut and hindgut are lined with different histological structures implies their different functions. It is possible that the midgut is involved in digestion, whereas the hindgut mainly in absorption, which is consistent with a previous study (Borror *et al*. 1989). Rectal papilla was not found in the epithelial layer of the hindgut.

### Female Reproductive System

**Figure 5:**
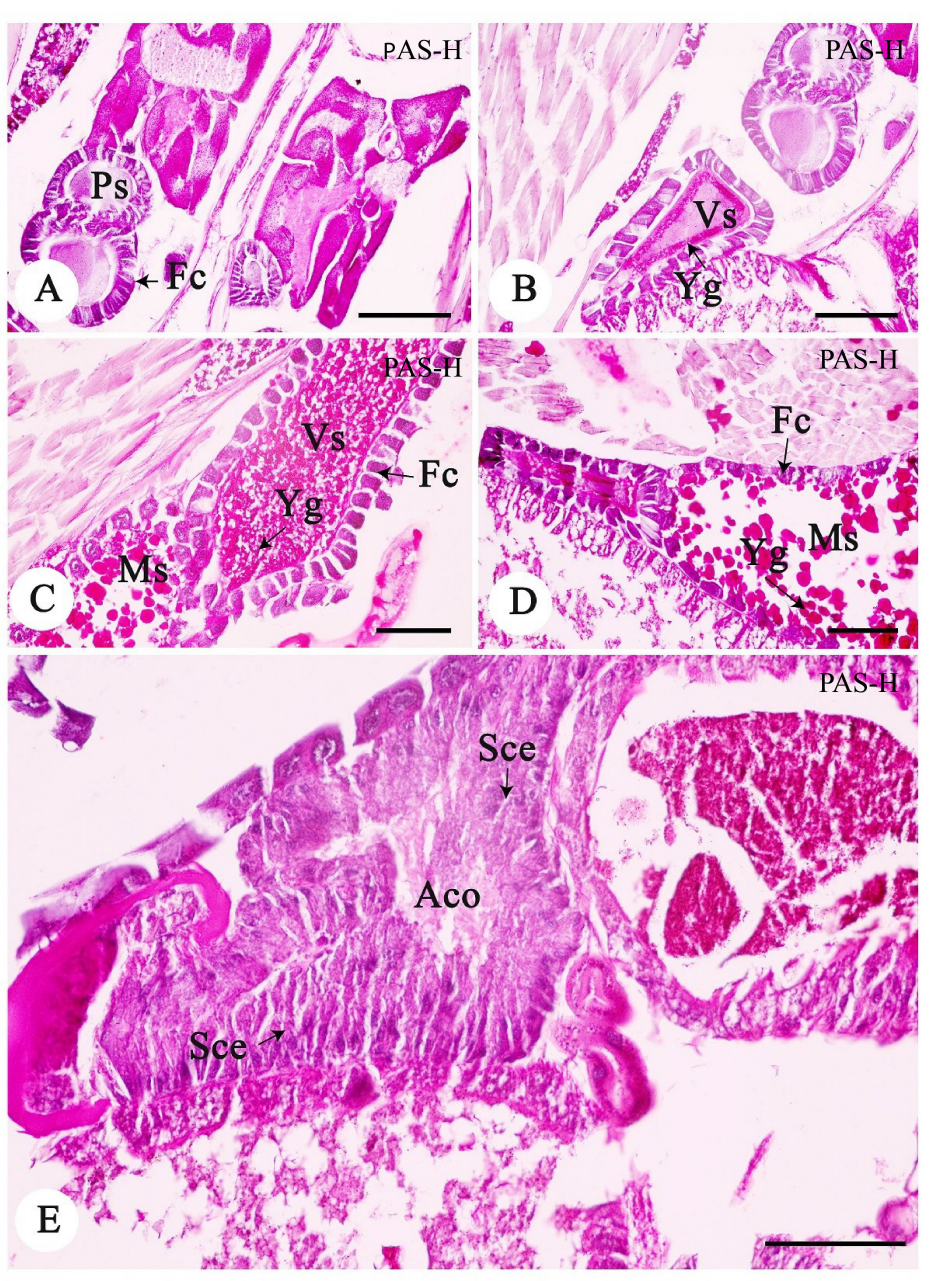
Light photomicrographs of the female reproductive system of *A. annandalei* containing oocytes in different developmental stages including Ps (A), Vs (B, C) and Ms (D). The accessory organ (Aco) of the reproductive system was histologically observed (E). *Notes*: Fc = follicular cell, Sce = stratified cuboidal epithelium, Yg = yolk granules. Scale bar A–F = 50 μm. Periodic acid-Schiff-hematoxylin (PAS-H).

The female reproductive system of *A. annandalei* was composed of two parts: a pair of ovaries and an oviduct, which was distributed along the midgut in the digestive system. Each ovary consisted of several ovarioles and considered to be the prognostic type (Fig. 5). The ovariole was enclosed by an external sheath, which could be divided into three regions including the anterior terminal filament, the germarium and the vitellarium.

An evaluation of the histological slides of the germinarium demonstrated the structure the anterior area of the ovariole. The oogonium was characterised by the large nucleus and small amounts of basophilic cytoplasm (Data not shown). In the vitellarium, oocytes at various development stages were present, which were classified into three stages [previtellogenic stage (Ps), vitellogenic stage (Vs) and mature stage (Ms)] (Figs. 5A–5D). Ps is the stage of increase in cell size. The cytoplasm of Ps oocytes was strongly basophilic (H&E method). The Ps oocytes were surrounded by a single layer of follicle cells (Fig. 5A). The Vs oocytes were differentiated from the Ps by their size. The cytoplasm of Vs oocytes started to form small spherical yolk granules (Fig. 5B). Each acidophilic yolk granule was positively reacted with PAS (Figs. 5B–5C). The follicular cells were clearly visible at this stage due to its height and the cytoplasm progressively changing to basophilic (Fig. 5C). Ms oocytes were the largest oocytes observed and had an irregular shape. The enlarged yolk granules were heterogeneously sized and dispersed throughout the ooplasm. Although the follicular cells in the Ms oocytes were lower in height than those of the Vs oocytes, the size of follicular cells in the Ms were thicker than those in the Vs (Fig. 5D). The rapid changes in the shape of follicular cells in the Ps to Vs stage were similar to those reported in *Periplaneta* and *Carausius* (Adderson 1964; Dutkowski & Grzelakowska 1965). These changes of follicular cells are likely related to the activity of the sex hormone in insects (Davey 1965). Follicular cells are the major production and conversion site of sex steroid hormones in insects.

Additionally, the accessory gland had elongated and basophilic structures (Fig. 5E). It was formed by the stratified cuboidal epithelium (Fig. 5E). The cell had a central nucleus with a strongly basophilic cytoplasm and connective tissues underneath.

## CONCLUSION

This histological study revealed the essential systems in *A. annandalei*, adding new information to the literature. It is important to note that the well-developed integument system and Malpighian tubule, together with the abundance of the respiratory organ, might be the adaption to the estuarine condition, by which *A. annandalei* can prevent the loss of water and metabolites. The information obtained from this pioneering study could lead to further depth studies in the future for this species and Genus *Asclepios*.
